# Nanohybrids of a MXene and transition metal dichalcogenide for selective detection of volatile organic compounds

**DOI:** 10.1038/s41467-020-15092-4

**Published:** 2020-03-10

**Authors:** Winston Yenyu Chen, Xiaofan Jiang, Sz-Nian Lai, Dimitrios Peroulis, Lia Stanciu

**Affiliations:** 1 School of Materials Engineering, Purdue University, West Lafayette, IN 47907 USA; 2 Birck Nanotechnology Center, Purdue University, West Lafayette, IN 47907 USA; 3 School of Electrical and Computer Engineering, Purdue University, West Lafayette, IN 47907 USA; 4 Department of Materials Science and Engineering, National Tsing Hua University, Hsinchu, 30013 Taiwan

**Keywords:** Materials science, Nanoscience and technology

## Abstract

Two-dimensional transition metal carbides/nitrides, known as MXenes, have been recently receiving attention for gas sensing. However, studies on hybridization of MXenes and 2D transition metal dichalcogenides as gas-sensing materials are relatively rare at this time. Herein, Ti_3_C_2_T_x_ and WSe_2_ are selected as model materials for hybridization and implemented toward detection of various volatile organic compounds. The Ti_3_C_2_T_x_/WSe_2_ hybrid sensor exhibits low noise level, ultrafast response/recovery times, and good flexibility for various volatile organic compounds. The sensitivity of the hybrid sensor to ethanol is improved by over 12-fold in comparison with pristine Ti_3_C_2_T_x_. Moreover, the hybridization process provides an effective strategy against MXene oxidation by restricting the interaction of water molecules from the edges of Ti_3_C_2_T_x_. An enhancement mechanism for Ti_3_C_2_T_x_/WSe_2_ heterostructured materials is proposed for highly sensitive and selective detection of oxygen-containing volatile organic compounds. The scientific findings of this work could guide future exploration of next-generation field-deployable sensors.

## Abstract (web summary)

Two-dimensional transition metal carbides and nitrides are promising for gas sensor applications. Here the authors report a nanohybrid-based wireless monitoring system with capabilities for selectivity and sensing for volatile organic compounds that are enhanced by heterojunction interfaces.

## Introduction

The significance of wearable and wireless technologies has been increasing rapidly with internet of things (IoTs)^1,2^, in which sensors are crucial components deemed necessary to collect massive amounts of information from surrounding environments. For example, volatile organic compounds (VOCs) are common air pollutants contributing to the formation of ground-level ozone and carcinogens, and are thus harmful to human health^3^. Therefore, it is important to develop a wireless-operating gas sensor for IoTs with rapid, selective, sensitive, and reversible detection of VOCs at room temperature.

Two-dimensional (2D) MXenes are generally produced by etching the intermediate A layers of a M_*n*+1_AX_*n*_ phase, where M, A, and X represent an early transition metal, A-group element, and carbon (or nitrogen) element (*n* = 1, 2, or 3), respectively^4^. Because of a combination of properties such as stable and easily tunable microstructure, high electrical conductivity, large chemically active surface, and adjustable hydrophilicity, low-dimensional MXenes and MXene-based nanocomposites have recently received considerable attention particularly to catalysis^5–7^, energy conversion/storage^8–10^, and biomedical applications^11–13^. Their application to gas sensor design, however, is rarely studied, and only focuses on pristine MXenes (Ti_3_C_2_T_x_, V_2_CT_x_, and Ti_2_CO_2_)^14–16^. On the other hand, 2D transition-metal dichalcogenides (TMDs) have been considered as promising sensing materials owing to their high surface-to-volume ratios, good adsorption properties, large number of active sites for redox reactions, and high surface reactivity^17,18^. Indeed, a significant number of literature reports have recently emerged regarding their integration into chemical sensors to detect various harmful gases, such as NH_3_, NO_2_, and VOCs^19^. Nevertheless, previous studies reported that gas-sensing devices using simply a 2D TMD material typically have a high electrical resistance and lack of selectivity and/or recovery to target analytes, especially at room temperature^19^, impeding their practical sensing applications to IoTs. To further improve their room-temperature sensing performance, 2D-TMDs have been fabricated vastly as alloy-based heterostructures (e.g., Mo(Se,S)_2_, WS_2x_Se_2-2x_)^20^, or incorporated with noble metallic nanoparticles (e.g., Ag, Nb, Pt)^21^, metal oxides (e.g., SnO_2_, ZnO, TiO_2_, Bi_2_O_3_)^22^, conducting polymers^23^, or graphene (or its derivatives)^24^.

In summary, both MXenes and TMDs proved to have device-beneficial physical and chemical properties (e.g., tunable band structures and microstructures), and each class of compounds has been individually studied extensively in recent years^25,26^. Incorporating TMD with MXene thus could be an effective strategy to further improve the room-temperature sensing performance of devices for VOCs. Besides hybrid MXene/TMD composites not being investigated for gas-sensing applications to date, most sensors reported in literature are fabricated by manual procedures, such as drop-casting, that can only be useful for laboratory scale production and suffer from lack of repeatability and precision. The lack of a pathway toward large-scale production is one of the main roadblocks toward bringing more sensors from the laboratory to the market, and one of the challenges we are addressing herein. The main approaches to synthesizing 2D functional materials are micromechanical exfoliation, liquid-phase exfoliation, ion intercalation-exfoliation, chemical vapor deposition, and wet chemical synthesis from molecular precursors^27–29^. Among these approaches, liquid-phase exfoliation appears to be the leading reliable, mass-production method for the wide-spread applications to gas-sensing devices. Such method avoids the use of dangerous air-sensitive reagents and undesired property changes. Likewise, inkjet deposition of exfoliated materials is also a facile and repeatable process to fabricate devices at large scale.

Herein, we report on the synthesis of MXene (Ti_3_C_2_T_x_) nanosheets and TMD (WSe_2_) nanoflakes both through liquid-phase exfoliation, inkjet printing of Ti_3_C_2_T_x_/WSe_2_ hybrid sensors for selective detection of oxygen-containing VOCs and a sensing mechanism for the enhanced oxygen-based VOCs detection with Ti_3_C_2_T_x_/WSe_2_ nanohybrids. Moreover, inkjet printing offers repeatability of electrode fabrication and reproducibility of the sensing measurements, and is one avenue toward large-scale manufacturing of electrochemical sensors. We report on a sensing material design for gas-sensing application based on integrating the merits of two components: Ti_3_C_2_T_x_ nanosheets, with effective charge transfer, and WSe_2_ nanoflakes, with abundant active sites for gas adsorption. The Ti_3_C_2_T_x_/WSe_2_ nanohybrids show a unique morphology with numerous heterojunction interfaces, consequently facilitating a selective detection of oxygen-containing VOCs. The Ti_3_C_2_T_x_/WSe_2_ hybrid sensors exhibit an over 12-fold increases in ethanol sensitivity compared to pristine Ti_3_C_2_T_x_ sensors. In addition, ultrafast response (9.7 s) and recovery (6.6 s) properties are achieved. We propose a sensing mechanism that is likely involved in the detection of oxygen-containing VOCs with Ti_3_C_2_T_x_/WSe_2_ heterojunctions and explains the observed enhanced sensing performance. A mass-production integration process (liquid-phase exfoliation and inkjet printing) and wireless operation of Ti_3_C_2_T_x_/WSe_2_ sensors at room temperature is demonstrated, thus opening an effective avenue for the development of high-performance sensing devices for next-generation IoTs.

## Results

### Sensor design

**Fig. 1:**
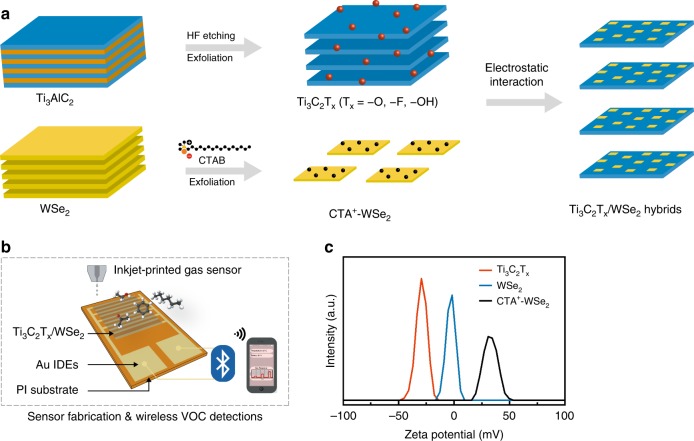
Ti_3_C_2_T_x_/WSe_2_ hybridization and sensor fabrication. **a** Schematic illustration of preparation processes for Ti_3_C_2_T_x_/WSe_2_ nanohybrids. **b** Schematic illustration of inkjet-printed gas sensors in detection of volatile organic compounds with a wireless monitoring system. **c** Zeta potential distributions of Ti_3_C_2_T_x_, WSe_2_, and CTA^+^-WSe_2_ dispersions.

Figure 1a illustrates the process flow of preparing (1) Ti_3_C_2_T_x_ nanosheets from sequential etching and exfoliating of Ti_3_AlC_2_ powders and (2) CTA^+^-WSe_2_ nanoflakes from CTAB functionalized WSe_2_ powders, followed by a solution mixing method to form Ti_3_C_2_T_x_/WSe_2_ nanohybrids. The Ti_3_C_2_T_x_/WSe_2_ nanohybrids were further prepared as ink for the inkjet printing and the fabrication of flexible VOC sensors operating at room temperature using a wireless monitoring system (Fig. 1b). As evidenced from Supplementary Fig. 1, the thickness of the inkjet-printed Ti_3_C_2_T_x_/WSe_2_ layers was controllable by the number of printing passes, with a thickness of ~60 nm per print pass.

The functional block diagram and photographic image of the flexible wireless sensor system (Supplementary Fig. 2), along with a homemade sensor testing system (Supplementary Fig. 3 and Supplementary Table 1), are detailed in Methods. The feasibility of these processes to form the Ti_3_C_2_T_x_/WSe_2_ nanohybrids via electrostatic attraction can be evidenced from 80%-potential measurements shown in Fig. 1c. The as-prepared Ti_3_C_2_T_x_ nanosheets are characterized by a negative charged surface with zeta potential of −29.5 mV, which could be attributed to the negatively charged −OH and −O species terminated on Ti_3_C_2_T_x_ surfaces (verified later by XPS). On the other hand, the zeta potential of the pristine WSe_2_ was only −1.5 mV. After exfoliation in CTAB aqueous solution, its surface charge was reversed, giving a substantial increase of zeta potential to +30 mV. This polarity reversion suggests that CTA^+^ cations indeed are effectively adsorbed on WSe_2_ nanoflakes, thereby facilitating the hybridization of Ti_3_C_2_T_x_ with WSe_2_ through electrostatic interaction.

The formation of as-etched Ti_3_C_2_T_x_ was further verified by XRD shown in Supplementary Fig. 4a. The removal of Al from Ti_3_AlC_2_ is revealed by the vanishing of (104) peak at 38.9°, along with the emergence of several peaks characteristics of Ti_3_C_2_T_x_^30^. Correspondingly, comparing the SEM micrographs in Supplementary Fig. 4b, c clearly reveals the transition from a bulk to an accordion-like structure upon the transformation of Ti_3_AlC_2_ to Ti_3_C_2_T_x_ nanosheets. The theoretical thickness of a Ti_3_C_2_T_x_ single layer is close to 1 nm^31^, and the MXene nanosheets tend to adsorb water and other molecules, which also add to the total thickness^32^. Indeed, the AFM height profile measured along the white line in Supplementary Fig. 4c shows that a representative Ti_3_C_2_T_x_ nanosheet has a thickness of ~1.5 nm, which can be regarded as MXene single layer^33^.

### Microstructure analysis of Ti_3_C_2_T_x_/WSe_2_ nanohybrid

**Fig. 2:**
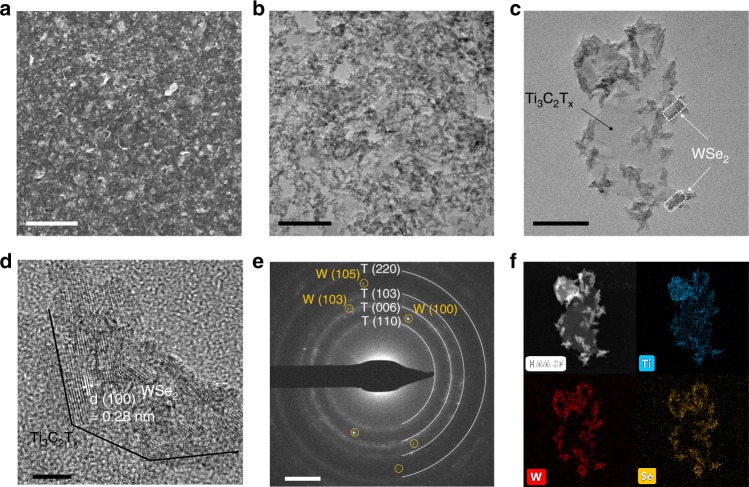
Microstructure analysis of Ti_3_C_2_T_x_/WSe_2_ nanohybrids. **a** SEM image of 2D Ti_3_C_2_T_x_/WSe_2_ nanohybrid film (scale bar, 2 μm). **b** Low magnification TEM image (scale bar, 200 nm), with **c** showing image of a single Ti_3_C_2_T_x_/WSe_2_ nanohybrid (scale bar, 100 nm). **d** High-resolution TEM image of Ti_3_C_2_T_x_/WSe_2_ nanohybrid (scale bar, 100 nm). **e** Selected area electron diffraction pattern of Ti_3_C_2_T_x_/WSe_2_ nanohybrids (scale bar, 2 nm^–1^). **f** HAADF-STEM image and corresponding elemental mapping of Ti, W, and Se for the Ti_3_C_2_T_x_/WSe_2_ nanohybrid showing a uniform decoration of WSe_2_ nanoflakes on Ti_3_C_2_T_x_ matrix.

SEM imaging, as demonstrated in Fig. 2a, reveals that the as-printed Ti_3_C_2_T_x_/WSe_2_ nanohybrids have a uniform surface morphology over a broad range of the samples area, in spite of the existence of a few pinholes. TEM imaging and diffraction analysis present further insight into the microstructures of the Ti_3_C_2_T_x_/WSe_2_ nanohybrids in Fig. 2b–d. TEM bright-field image (Fig. 2b) shows that WSe_2_ nanoflakes appear to disperse homogeneously on the Ti_3_C_2_T_x_ matrix. As WSe_2_ has a large atomic weight, a clear differentiable contrast in this bright-field micrograph is observed, with the darker WSe_2_ nanoflakes landed on the brighter Ti_3_C_2_T_x_ nanosheets. Such distribution is more obvious in higher magnification TEM image of a single Ti_3_C_2_T_x_/WSe_2_ nanohybrid in Fig. 2c, showing (a) uniform decoration of several even sized (typical < 30 nm) WSe_2_ nanoflakes on the Ti_3_C_2_T_x_ scaffolds which have a typical size of ~300 nm and (b) the hybridization process forming a numerous heterojunction interfaces that may benefits the gas-sensing performance. The dynamic light scattering measurements (Supplementary Fig. 5) indicate that Ti_3_C_2_T_x_/WSe_2_ nanohybrids exhibit an average particle size of 350 ± 100 nm, closely consistent with the TEM imaging analysis.

As demonstrated by the high-resolution TEM image in Fig. 2d, WSe_2_ nanoflakes with a lattice fringe of 0.28 nm were distributed on the edges of the Ti_3_C_2_T_x_ nanosheets, which corresponds to the (100) plane of hexagonal WSe_2_^34^. Moreover, the associated selected area electron diffraction pattern in Fig. 2e reveals various diffraction rings (denoted as T), indexed as from the matrix of the hexagonal Ti_3_C_2_T_x_ nanosheets. Meanwhile, some diffraction spots (denoted as W) coexist with the continuous rings attributed to the adsorbed WSe_2_ nanoflakes. To investigate further the elemental distribution of the Ti_3_C_2_T_x_/WSe_2_ nanohybrid structure, high-angle annular dark-field−scanning transmission electron microscopy (HAADF-STEM) imaging and the energy-dispersive X-Ray elemental mapping were carried out, and Fig. 2f presents a representative result, indicating a uniform distribution of Ti, W, and Se within the hybrid.

### Chemical composition of Ti_3_C_2_T_x_/WSe_2_ nanohybrids

**Fig. 3:**
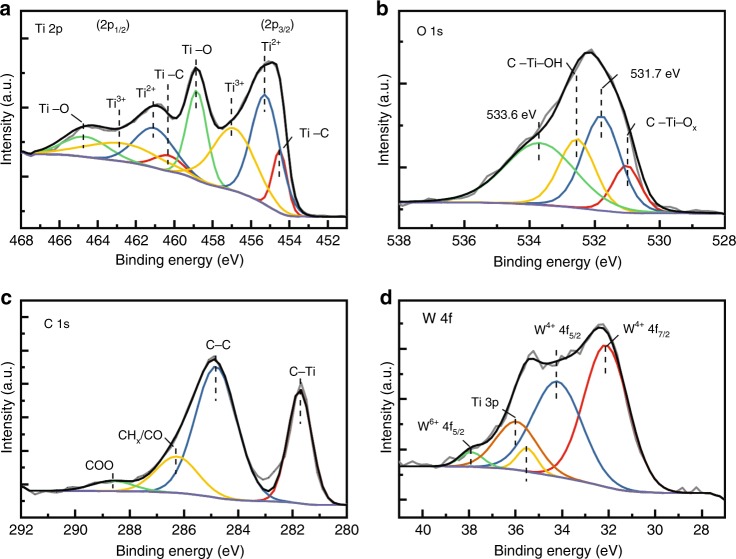
Chemical composition of Ti_3_C_2_T_x_/WSe_2_ nanohybrids. High-resolution XPS spectra of **a** Ti 2p, **b** O 1s, **c** C 1s, and **d** W 4f from Ti_3_C_2_T_x_/WSe_2_ nanohybrids, showing chemical components and structures of Ti_3_C_2_T_x_/WSe_2_ nanohybrids.

Figure 3a–d shows a set of high-resolution XPS spectra (Ti 2p, O 1s, C 1s, and W 4f) taken from Ti_3_C_2_T_x_/WSe_2_ nanohybrids. Interpreting these spectra can identify the chemical structure of WSe_2_ nanoflakes and Ti_3_C_2_T_x_ nanosheets, as well as the successful fabrication of Ti_3_C_2_T_x_/WSe_2_ nanohybrids. The Ti 2p spectrum (Fig. 3a) was fitted with a fixed area ratio of 2:1 and a doublet separation of 5.8 eV comprising four doublets (Ti 2p_3/2_ and Ti 2p_1/2_). The binding energies of Ti 2p_3/2_ for Ti−C, Ti^2+^, Ti^3+^, and Ti−O are 454.5, 455.3, 456.9, and 458.9 eV, respectively, in agreement with previous XPS studies^35–37^. Herein, the fresh Ti_3_C_2_T_x_ scaffold was successfully fabricated and it was significantly different from the oxidized MXene where only Ti−O peaks were observed (Supplementary Fig. 6)^37^. The O 1s spectrum in Fig. 3b can be deconvoluted into four peaks centered at 530.9, 531.7, 532.6, and 533.6 eV, corresponding to surface species of C−Ti−O_x_, C−Ti−OH, adsorbed oxygen and H_2_O, respectively^38,39^. This finding confirms that the surface of the Ti_3_C_2_T_x_ nanosheet indeed is terminated by an abundance of functional groups, facilitating its hybridization with WSe_2_. The C 1s spectrum in Fig. 3c was deconvoluted to four peaks centered at 281.7, 284.8, 286.3, and 288.6 eV, corresponding to C−Ti, C−C, CH_x_/CO and COO, respectively^36^. The existence of WSe_2_ nanosheets is also confirmed by the high-resolution XPS spectrum in Fig. 3d, which shows two main characteristic peaks of W^4+^ at 32.2 eV (W 4f_7/2_) and 34.3 eV (W 4f_5/2_).

### Gas-sensing performance of WSe_2_ decorated MXene sensors

The sensing performance of Ti_3_C_2_T_x_ decorated with various amounts of WSe_2_ (2 and 4 wt%) toward 40-ppm ethanol was first examined using individual Ti_3_C_2_T_x_ and WSe_2_ sensors as references. A detailed account of the results is presented in Supplementary Information (Supplementary Fig. 7a–d and Supplementary Note 2). The response of both Ti_3_C_2_T_x_ and WSe_2_ sensors is inferior to that of Ti_3_C_2_T_x_ loaded with a moderate amount of WSe_2_ nanoflakes (2 wt%), the latter showing the strongest and fastest response toward ethanol. It can be observed that the ethanol response decreases as the WSe_2_ loading increases from 2 to 4 wt%, which is attributed to the excessive number of WSe_2_ nanoflakes blocking heterojunctions of Ti_3_C_2_T_x_/WSe_2_ hybrids (proposed as the major adsorption sites), as also observed from HAADF-STEM images (Supplementary Fig. 7f, g). Their electrical noise was further determined by measuring the response fluctuation during sensor exposure to air. The electrical noise levels of WSe_2_, Ti_3_C_2_T_x_, and Ti_3_C_2_T_x_/WSe_2_ (2 wt%) sensors were ~1, 0.08, and 0.15%, respectively; the high noise level registered for WSe_2_ limits its use for high-performance sensors and stems from its high electrical resistance. Moreover, sheet resistance (±1σ; *N* = 3) of the pristine WSe_2_ films was 26.3 ± 5.2 MΩ per square, while that of the Ti_3_C_2_T_x_ films loaded with 2 wt% WSe_2_ dramatically reduced to 3.3 ± 0.5 kΩ per square; this is more than four orders of magnitude decrease in sheet resistance (equivalent increase in the electrical conductivity), owing to the hybridization of WSe_2_ with Ti_3_C_2_T_x_ with high metallic conductivity. Notably, the sensor based on Ti_3_C_2_T_x_/WSe_2_ nanohybrids not only exhibits the highest gas response, but also displays a low-electrical noise, further cementing the finding that the hybridization of MXenes to a TMD material yields superior VOC sensing performance in conductometric devices compared to individual MXene and TMD.

The thickness of sensing films deposited on electrodes in such devices is another key factor affecting performance^40,41^. Thus, gas-sensing performance of Ti_3_C_2_T_x_/WSe_2_ films with thicknesses of 60, 120, and 180 nm was examined by comparing their response curves toward the detection of 40-ppm ethanol and acetone (Supplementary Fig. 8). The response of the Ti_3_C_2_T_x_/WSe_2_ sensors to both VOCs decreased dramatically with increasing film thickness; the ethanol response substantially decreased from −9.2% to only −0.7% as film thickness increased from 60 nm to 180 nm. This is most likely because thickness increase of Ti_3_C_2_T_x_/WSe_2_ films lowers the surface-to-volume ratio of the Ti_3_C_2_T_x_/WSe_2_ channel, impeding gas uptake and transport within the film.

**Fig. 4:**
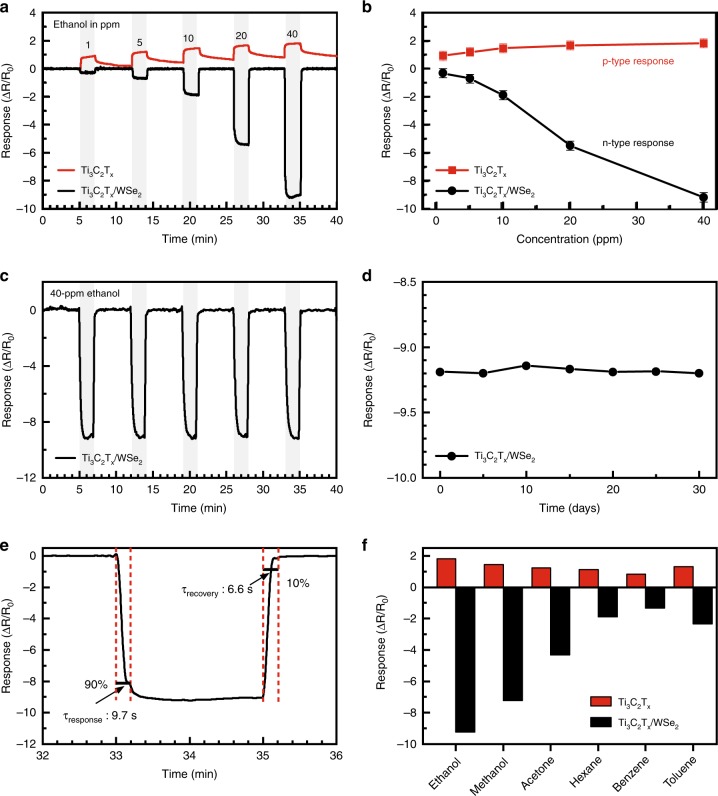
Sensing characteristics of MXene-based VOC sensors. **a** Real-time sensing response of Ti_3_C_2_T_x_ and Ti_3_C_2_T_x_/WSe_2_ gas sensors upon ethanol exposure with concentrations ranging from 1 to 40 ppm. **b** Comparison of gas response as a function of ethanol gas concentrations for Ti_3_C_2_T_x_ and Ti_3_C_2_T_x_/WSe_2_ sensors. **c** Cycling performance of Ti_3_C_2_T_x_/WSe_2_ gas sensors in response to ethanol at 40 ppm level. **d** Long-term stability of response over a month under 40 ppm of ethanol for Ti_3_C_2_T_x_/WSe_2_ sensor. **e** Response and recovery times calculated for 40 ppm of ethanol. **f** Selectivity test of the Ti_3_C_2_T_x_ and Ti_3_C_2_T_x_/WSe_2_ sensors upon exposure to various VOCs at 40 ppm.

**Fig. 5:**
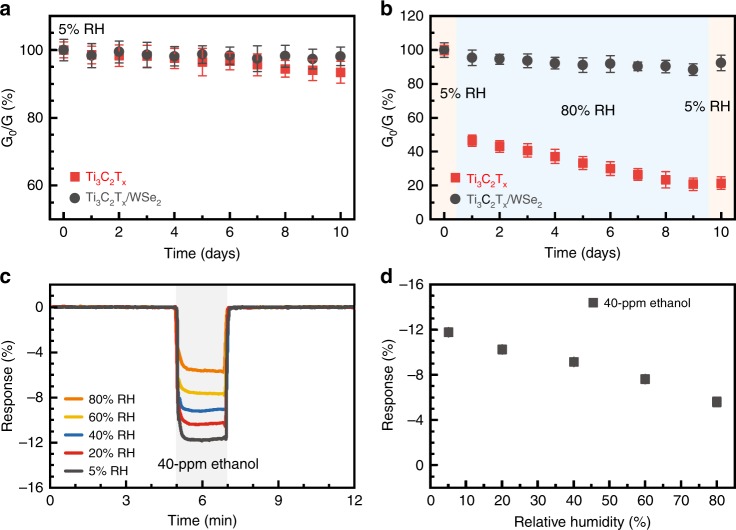
Environmental stability of Ti_3_C_2_T_x_ and hybrid Ti_3_C_2_T_x_/WSe_2_ films. Changes in electrical conductance of pristine Ti_3_C_2_T_x_ and hybrid Ti_3_C_2_T_x_/WSe_2_ sensors under **a** 5% RH and **b** alternative RHs of 5 and 80% over 10 days. **c** Evolution of responses of Ti_3_C_2_T_x_/WSe_2_ sensors to 40 ppm of ethanol under various RHs and **d** the sensing responses as a function of RHs from 5 to 80%.

Hereafter, we have fabricated dozens of sensors made of 60-nm-thick Ti_3_C_2_T_x_/WSe_2_ electrode; three sensors were subjected to a variety of further sensing tests in parallel each run, using a Ti_3_C_2_T_x_ sensor as reference. The plots presented in Fig. 4 are typical of these measurements. Figure 4a presents sensing properties of pristine Ti_3_C_2_T_x_ and hybrid Ti_3_C_2_T_x_/WSe_2_ sensors upon exposure to ethanol vapors over a wide range of concentrations from 1 to 40 ppm. The Ti_3_C_2_T_x_ sensor shows a positive but relatively small increase of resistance to ethanol (p-type sensing behavior), indicating that the charge carrier transport channel is impeded by the adsorption of ethanol molecules. This positive response is ascribed to the metallic conductivity of Ti_3_C_2_T_x_, where gas adsorption reduces the number of charge carriers (electrons), resulting in an increase of channel resistance^42^. The unrecoverable response of the Ti_3_C_2_T_x_ sensor is observed by a slight upward drift of the baseline because of the incomplete gas desorption from Ti_3_C_2_T_x_ caused by chemisorption of ethanol^43^. Interestingly, the Ti_3_C_2_T_x_/WSe_2_ sensor displays a negative variation of resistance in the presence of ethanol (n-type sensing behavior) in Fig. 4a, implying that the Ti_3_C_2_T_x_/WSe_2_ heterostructured sensor is dominated by different sensing mechanism compared to pristine Ti_3_C_2_T_x_. The responses of the Ti_3_C_2_T_x_ and Ti_3_C_2_T_x_/WSe_2_ sensors with concentration variations of ethanol are shown in Fig. 4b. Notably, standard deviations of the measured response values for the various ethanol concentrations were only 3.7% at most. Such small deviations suggest that the inkjet printing used here indeed offers high repeatability of electrode fabrication, thus giving low device-to-device variations. The response was almost linear and the sensitivity of the sensors here was calculated as the slope (response/ppm), showing a significant increase of sensitivity from 0.02 to 0.24 (12-fold) by the hybridization of Ti_3_C_2_T_x_ and WSe_2_. The response of the Ti_3_C_2_T_x_/WSe_2_ sensor to ethanol does not reach a saturated state toward 40 ppm of ethanol, indicating that the sensor has a strong ability to detect ethanol molecules over a wide range of concentrations. These enhancements are likely due to the formation of Ti_3_C_2_T_x_/WSe_2_ heterojunctions, providing not only fast electron transport but also acting as effective catalysts due to its appropriate chemical potential^44^. Thus, hybridizing WSe_2_ with Ti_3_C_2_T_x_ significantly enhances the gas-sensing performance and the detailed sensing mechanism will be discussed later in Fig. 5.

Figure 4c depicts the exposure of the Ti_3_C_2_T_x_/WSe_2_ sensor to 40-ppm ethanol for five consecutive cycles, demonstrating its repeatable, fast gas response and recovery. Moreover, the long-term stability of Ti_3_C_2_T_x_/WSe_2_ sensor was evaluated upon exposure to 40-ppm ethanol for a month at interval of 5 days (Fig. 4d). The response remained at around −9.2% over a month period, indicating a good long-term stability of the Ti_3_C_2_T_x_/WSe_2_ sensor. This suggests that our hybridization process might also be an effective strategy to overcome the oxidation of MXenes^37,45^. Fig. 4e shows response/recovery properties of the Ti_3_C_2_T_x_/WSe_2_ sensor toward 40 ppm of ethanol. The response time was defined as the time taken to reach 90% of the maximum gas response after the introduction of a VOC analyte. The recovery time was defined as the time taken to return to 10% of the minimum gas response after the removal of the target analyte. The Ti_3_C_2_T_x_/WSe_2_ sensor demonstrated an ultrafast response (9.7 s) and recovery (6.6 s) at room temperature. The enhancement of gas-sensing performance could be attributed to the heterojunction formation that (a) effectively accelerates the transport of electrons and (b) serves as catalyst lowering the activation energy of gas analytes^44,46^.

To understand further the benefit of hybridizing Ti_3_C_2_T_x_ with WSe_2_ in the detection of various VOCs, the pristine Ti_3_C_2_T_x_ and Ti_3_C_2_T_x_/WSe_2_ sensors were exposed to 40 ppm of various oxygen-containing VOCs: ethanol (C_2_H_5_OH), methanol (CH_3_OH), and acetone (CH_3_COCH_3_) and carbon-based VOCs: hexane (C_6_H_14_), benzene (C_6_H_6_), and toluene (C_6_H_5_CH_3_); their response values are presented in Fig. 4f. For each of the individual target analytes, the Ti_3_C_2_T_x_ sensor shows a positive, smaller response value, while the Ti_3_C_2_T_x_/WSe_2_ sensor shows a negative and much higher response value. In general, the carbon-based molecules’ (benzene, toluene and hexane) interaction with sensing surfaces is minimal, which results in a relatively small resistance variation for both sensors. Both Ti_3_C_2_T_x_ and Ti_3_C_2_T_x_/WSe_2_ sensors exhibit a slightly higher response to toluene and hexane, as compared to benzene, owing to the presence of their methyl groups, which induces dipole scattering^47^. On the other hand, the Ti_3_C_2_T_x_/WSe_2_ sensor exhibits an enhancement in terms of selectivity and sensitivity toward the sensing of oxygen-containing molecules (ethanol, methanol, and acetone). The sensing behavior of Ti_3_C_2_T_x_/WSe_2_ hybrid sensor to oxygen-containing molecules is rather complicated and has not been well explored.

### Environmental and mechanical stability of Ti_3_C_2_T_x_-based sensors

A serious challenge for Ti_3_C_2_T_x_ nanosheets being used as functional coatings or truly useful device materials is their high susceptibility to environmental degradation under humid atmosphere or in aqueous solution^33,48,49^. We hypothesize that hybridization with WSe_2_ could help overcome this challenge. To test this hypothesis, we selected water vapor over a wide range of relative humidity (RH) from 5 to 80% as an interference component and tested the environmental stability of the hybrid Ti_3_C_2_T_x_/WSe_2_ film using pristine Ti_3_C_2_T_x_ film as a control. A full account of results is presented in Fig. 5a−d. Figure 5a shows small changes in electrical conductance of Ti_3_C_2_T_x_ and Ti_3_C_2_T_x_/WSe_2_ films under low humidity (5%) over a period of 10 days, indicating that both films are quite stable in the dry environment. However, after storage in the humid environment with alternative RHs of 5 and 80% over 10 days, electrical conductance of the Ti_3_C_2_T_x_ dramatically decreased to 21% of its original value; by contrast, the Ti_3_C_2_T_x_/WSe_2_ remained 88% of initial conductance after exposure to 80% of RH, and recovered to 92% in dry environment (Fig. 5b).

To evaluate the effect of humidity levels on gas-sensing performance of the hybrid Ti_3_C_2_T_x_/WSe_2_ sensor, we measured its response to 40 ppm of ethanol under various RH levels from 5 to 80%. As revealed by Fig. 5c, d, the response of the Ti_3_C_2_T_x_/WSe_2_ sensor changes from −12 to −6.1% as the humidity level increases from 5 to 80%, indicating that although there is a humidity effect, the hybrid sensor is still functioning well in high humidity environment. The decrease in response is attributed to partial occupancy of water molecules on the sensing sites, causing a decrease in sensing performance^50^. The results suggest that MXenes indeed tend to oxidize in a humid environment, but adequate loading of TMD nanoflakes to edges of MXene nanosheets provides an effective strategy to block the direct interaction of H_2_O with MXenes, which thus is an approach to promoting MXene materials for real-life applications.

**Fig. 6:**
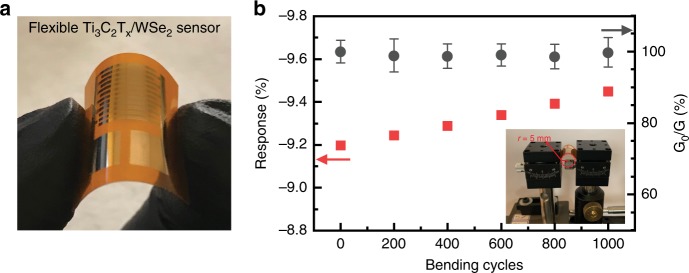
Mechanical stability of hybrid Ti_3_C_2_T_x_/WSe_2_ films. **a** Photograph of a flexible Ti_3_C_2_T_x_/WSe_2_ nanohybrid sensor. **b** Changes in ethanol sensing response and electrical conductance as a function of bending cycles.

The stability of conductance and sensing response of the fully inkjet-printed flexible Ti_3_C_2_T_x_/WSe_2_ (wireless) sensor against mechanical bending was also investigated demonstrating its potential application to IoTs, using ethanol as a target analyte. Figure 6a displays a photograph of the flexible gas sensor. As shown in Fig. 6b, even after 1000 cycles of a bending test with a bending radius of 5 mm, the response of the sensor to 40 ppm of ethanol does not decay; instead, it increased slightly probably due to the creation of bending-induced reactive sensing sites, such as microcracks and wrinkles by the strain force^34^. Moreover, the electrical conductance of the sensor was rather stable even after 1000 bending cycles (Fig. 6b), indicating good flexibility and high mechanical strength of the Ti_3_C_2_T_x_/WSe_2_ sensor. The retaining of the conductance level on the baseline of the sensor suggests that prolonged bending does not have a negative impact on the sensing properties of the Ti_3_C_2_T_x_/WSe_2_ sensor.

### Enhanced sensing mechanism for Ti_3_C_2_T_x_/WSe_2_ nanohybrids

**Fig. 7:**
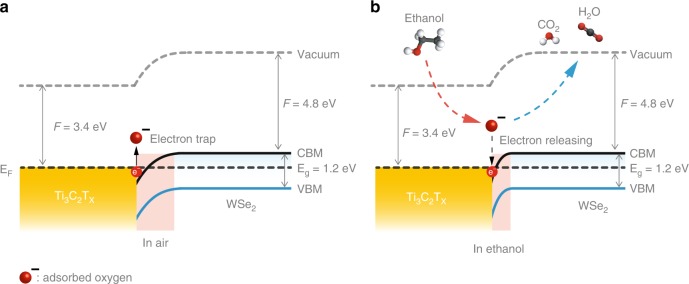
Enhanced sensing mechanism of Ti_3_C_2_T_x_/WSe_2_ heterostructure. Energy-band diagram of the Ti_3_C_2_T_x_/WSe_2_ in **a** air and **b** ethanol, showing the variation of the depletion layer with interaction between adsorbed oxygen species and ethanol molecules.

Herein, we propose a sensing mechanism for the enhanced oxygen-based VOCs detection with Ti_3_C_2_T_x_/WSe_2_ nanohybrids. As shown in Fig. 7a, the band structure of Ti_3_C_2_T_x_/WSe_2_ nanohybrids, with a partially occupied band crossing the Fermi level, offers a good catalytic effect for enhancements of sensing reactions because the highly conductive Ti_3_C_2_T_x_ nanosheets readily supplies a flow of electrons to WSe_2_^44^. In fresh air, the electrons were trapped by adsorbed oxygen species (O_2_^−^ and O^−^) owing to its electron-deficient nature, thus creating a depletion layer. Upon exposure to oxygen-based VOCs (Fig. 7b), the adsorbed active oxygen species react with ethanol molecules subsequently forming volatile gases (CO_2_ and H_2_O) and releasing electrons back to the conduction band, thereby resulting in a reduction in the depletion layer and resistance of the sensor (n-type sensing behavior in Ti_3_C_2_T_x_/WSe_2_ channels). Notably, Ti_3_C_2_T_x_/WSe_2_ nanohybrids significantly increase adsorbed oxygen species (in turn trap more electrons) because of the numerous heterojunction interfaces formed (as shown from TEM imaging), resulting in a large number of captured electrons released back to the Ti_3_C_2_T_x_/WSe_2_ channel and thus significantly improving the sensing response and selectivity in detection of oxygen-containing VOCs.

A variety of strategies have been conducted to enhance the gas-sensing performance of 2D TMD, including incorporation of metallic nanoparticles, semiconducting metal oxides, conducting polymers or carbon-based materials^20–24^. The Ti_3_C_2_T_x_/WSe_2_ heterostructured sensors examined here are compared with other representative 2D TMD-based hybrid sensors listed in Supplementary Table 2. Our integration of the solution processing method in the synthesis of a gas-sensing material, namely Ti_3_C_2_T_x_/WSe_2_ nanohybrid, was successful toward the fabrication of flexible VOC sensors using inkjet printing. Importantly, the Ti_3_C_2_T_x_/WSe_2_ sensors reported herein show either a lower detection limit than most other ethanol sensors, or a higher sensitivity at room temperature. Moreover, we demonstrated ultrafast response/recovery properties, which were not reported in most other publications on the detection of ethanol. In addition, the low-electrical-noise Ti_3_C_2_T_x_/WSe_2_ nanohybrid offers exceptional stability and durability against prolonged mechanical bending and environmental testing, along with the manufacturability of a sensor platform demonstrated, offer a unique opportunity for real-sensing applications to IoTs. For example, the high electrical noise values demonstrated by individual transition metal dichalcogenide materials limit the actual use of such sensors even when laboratory-based performance is adequate. The integration of MXene/TMD hybrid sensors with the features of Bluetooth wireless communication, flexibility and durability shed light on the development of next-generation field-deployable sensor devices suitable for IoT applications.

## Discussion

We have reported Ti_3_C_2_T_x_/WSe_2_ nanohybrids, fabricated through a facile surface-treating and exfoliation-based process, as sensing materials incorporated in an inkjet-printing and wirelessly-operating sensor for the detection of a variety of VOCs at room temperature. Inkjet printing offers repeatable fabrication of Ti_3_C_2_T_x_/WSe_2_ sensors, thus giving low device-to-device variations and reproducibility of the sensing measurements. Compared with the sensors made of pristine Ti_3_C_2_T_x_ and pristine WSe_2_, the Ti_3_C_2_T_x_/WSe_2_ hybrid sensor exhibits a 12-fold increase in ethanol sensitivity, low-electrical noise, sound selectivity, and ultrafast response/recovery properties. Moreover, this study sheds light on the hybridization of MXenes with TMDs as sensing materials overcoming the notorious instability and oxidization tendency of individual MXenes, which thus is an approach to promoting MXene materials for real-life applications. The enhancement of the sensing performance to oxygen-containing VOCs is likely due to the numerous heterojunction interfaces formed by Ti_3_C_2_T_x_/WSe_2_ nanohybrids and its sensing mechanism is proposed. Thus, the flexible sensors reported here have a high potential for use as practical gas-sensing devices for IoTs. We anticipate that the hybridizing approach of this work would be extended to other 2D MXene materials for sensing applications.

## Methods

### Preparation of Ti_3_C_2_T_x_ nanosheets, WSe_2_ nanoflakes, and Ti_3_C_2_T_x_/WSe_2_ inks

A total of 5 g of Ti_3_AlC_2_ powders (particle size < 40 μm, Carbon-Ukraine Ltd.) were etched in 100 mL of hydrofluoric (30%) aqueous solution and stirred for 24 h at room temperature to remove Al atoms from the Ti_3_AlC_2_ powders, Then, the Ti_3_C_2_T_x_ powders were washed via centrifugation several times with deionized water until the pH value of the supernatant reached around 6. The sediment was collected and rewashed with deionized water by vacuum filtration using difluoride membrane with 0.22 μm pore size (Durapore, Millipore), subsequently dried in a vacuum oven at 60 °C for 8 h. The fabrication of Ti_3_C_2_T_x_ nanosheets was performed by sonicating 200 mg of Ti_3_C_2_T_x_ powders in 50 mL of deionized water with ultrasonic bath (Branson, CPX2800H) for 1 h, and centrifuged at 3500 rpm for 1 h to separate the Ti_3_C_2_T_x_ layers. To avoid restacking of the nanosheets caused by the thermal energy released during sonication processing, the bath temperature was controlled at 4 °C. The supernatant containing delaminated Ti_3_C_2_T_x_ nanosheets was collected for further hybridization. The concentration of Ti_3_C_2_T_x_ dispersion was measured by vacuum filtering the colloidal solution (5.1 ± 0.1 mg mL^−1^). A total of 200 mg of WSe_2_ powders (99% purity) from Sigma-Aldrich were dispersed in 20 mL of 1% cetyltrimethylammonium bromide (CTAB) aqueous solution followed by sonication at 4 °C for 10 h in an ultrasonic bath. As proven by zeta potential measurements, this treatment results in the adsorption of CTA^+^ cations onto WSe_2_ nanoflakes. The functionalized dispersion was sequentially centrifuged at 2000 rpm and 5000 rpm, which narrowed down the size distribution^51^. The supernatant containing CTA^+^-WSe_2_ nanoflakes (1.3 ± 0.1 mg mL^−1^) was collected for further reaction. A total of 10 mL of Ti_3_C_2_T_x_ (as the hosting matrix of the nanohybrid) aqueous solution were added into CTA^+^-WSe_2_ solution at 60 °C and stirred for 2 h to form a Ti_3_C_2_T_x_/WSe_2_ hybrid by electrostatic interaction. Ti_3_C_2_T_x_, WSe_2_, and Ti_3_C_2_T_x_ nanosheets mixed with 2 and 4 wt% of WSe_2_ nanoflakes were prepared for sensing performance evaluation. Glycerol was then added to the dispersions with an optimum weight ratio of 1:3, achieving a required viscosity for inkjet printing, which was typically around 10 cP^52^.

### Fabrication of inkjet-printed Ti_3_C_2_T_x_/WSe_2_ sensors

Nanogold ink (UTDAu40) from UT Dots, Inc. was printed by a commercial Dimatix DMP-2850 inkjet printer on polyimide substrates containing six pairs of gold interdigitated electrodes (IDEs) with a total active electrode area of 8 mm × 8 mm. Sensing layers of Ti_3_C_2_T_x_, WSe_2_ (as references) and Ti_3_C_2_T_x_/WSe_2_ were printed, respectively, on the electrode surface of the flexible substrates, which were placed on a vacuum-heated platen and kept at 60 °C to achieve a stable drying rate.

### Characterization and gas-sensing measurements

Surface morphology and crystallinity of the Ti_3_C_2_T_x_/WSe_2_ nanohybrids were examined by scanning electron microscopy (SEM; S-4800, Hitachi), transmission electron microscopy (TEM), high-angle annular dark-field scanning transmission electron microscopy (HAADF-STEM; Talos 200×, FEI), and X-ray diffractometry (XRD; X’Pert Pro, Panalytical) operated at 45 kV and 40 mA using Cu K_α_. X-ray photoelectron spectroscopy (XPS; PHI 5000 Versaprobe, ULVAC-PHI) was used to investigate the chemical components and chemical bonding structures of the Ti_3_C_2_T_x_/WSe_2_ nanohybrids. Atomic force microscopy (AFM; Dimension 3100, Veeco) was used in tapping mode to measure film thickness and the profile of the nanosheets. Electrical sheet resistance of the films was measured using a Jandel four-point probe system. The measurements were taken from four different spots on the sample surface and the average values were presented. Dynamic light scattering (Zetasizer Nano ZS, Malvern Instruments) was used to examine the zeta potential and particle size distribution of the materials in aqueous solutions.

Gas-sensing measurements were performed in a homemade sensor testing system (Supplementary Note 1, Supplementary Fig. 3, and Supplementary Table 1)^53^. Briefly, the sensors were placed in a Teflon chamber with gas inlet and outlet pipelines. Mass flow controllers (5850E, Brooks Instruments) were used to control the concentrations of VOC analytes, by adjusting the flow rates of VOC analytes and dilution gas (dry air), with a total flow rate fixed at 500 ml/min. The bubbler containing VOC analytes was set at a controlled temperature to maintain a stable vapor pressure. The gas concentrations were calibrated with a commercial VOC sensor (Honeywell, ToxiRAE Pro PID). Humidity interference tests were performed by introducing a VOC analyte gas into saturated salt solution and the relative humidity (RH) was monitored with a commercially available humidity sensor (HDC 2010, Texas Instruments).

The response of the sensor is defined as:

1 $${\mathrm{Response}}\left( \% \right)\,{\mathrm{ = }}\,\frac{{R_g - R_0}}{{R_0}}{\mathrm{ \times 100\% = }}\,\frac{{\Delta R}}{{R_0}}{\mathrm{ \times 100\% }}$$

where *R*_*g*_ and *R*_*0*_ represent electrical resistances of the sensors in the presence of VOC analytes and dry air, respectively.

### Wireless gas-sensing system

Electrical signals of the gas sensor were detected by a wireless reading system, and the corresponding functional block diagram and photographic image of the flexible sensor system (connected to the wireless reading system) are displayed in Supplementary Fig. 2. The wireless reading system consists of a wireless transceiver (nRF52832 SoC), a dual readout interface, analog-to-digital converter (ADC; NAU7802), and a highly accurate humidity/temperature sensor (HDC2010) from Texas Instruments. The transceiver features an ultralow-power 32-bit ARM Cortex-M4F microprocessor with a built-in radio that operates in the 2.4 GHz ISM band, and 512 kB flash memory for data logging when the sensor is disconnected from the network. Instant variations of the signals from gas sensors are detected by the analog-to-digital converter and converted to corresponding digital signals by the microprocessor such that they are wirelessly transmitted to a mobile device through the Bluetooth Low Energy (BLE). The dual readout interface was implemented by a 2-to-1 analog multiplexer that connects into a Wheatstone bridge with a digital potentiometer controlled by the microprocessor via I^2^C bus. During operation, the digital potentiometer is adjusted based on the ADC reading to match the resistance of the sensor as close as possible. This readout interface supports dual input with extreme high precision and minimal power consumption. The system is powered by a coin cell battery (cr2032).

## Supplementary information

Supplementary Information

## Data Availability

The data that support the findings of this study are available from the corresponding author upon reasonable request.
