# Supplementary material for: Nanohybrids of a MXene and transition metal dichalcogenide for selective detection of volatile organic compounds
Source: Nat Commun. 2020 Mar 10;11:1302. doi: 10.1038/s41467-020-15092-4 (PMC7064528; doi:10.1038/s41467-020-15092-4)
Supplement: Supplementary file 1 — Supplementary Information [file 41467_2020_15092_MOESM1_ESM.pdf]

Supplementary Information

Nanohybrids of MXene and Transition Metal Dichalcogenide for Selective Detection of Volatile Organic Compounds

Chen et al.

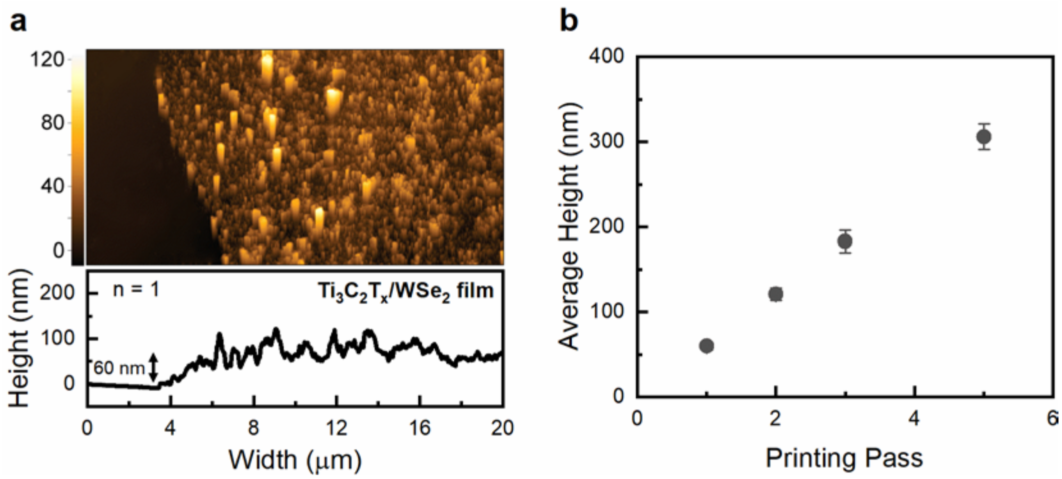

Supplementary Fig. 1 Thickness measurements of inkjet-printed $Ti_3C_2T_x/WSe_2$ films. a AFM imaging and corresponding height profile typical of $Ti_3C_2T_x/WSe_2$ gas-sensing films for one printing pass. **b** Average film thicknesses derived from AFM for as-printed $Ti_3C_2T_x/WSe_2$ films versus number of printing passes of 1, 2, 3, and 5, indicating a thickness of approximately 60 nm for one printing pass.

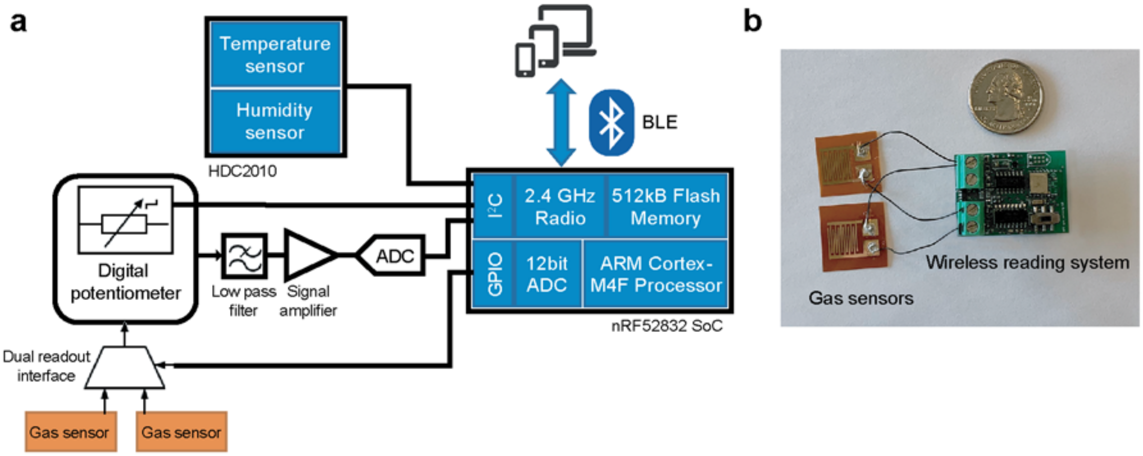

Supplementary Fig. 2 Wireless sensor system. a Functional block diagram. **b** Image of a wireless-reading flexible sensor system.

Supplementary Note 1: Gas-sensing system and controls of humidity and flow rates/concentrations of VOCs. Supplementary Fig. 3 shows the schematic setup of a homemade gas-sensing system used in this study. To monitor the response of the sensors to various VOCs (ethanol, methanol, acetone, hexane, benzene, and toluene), the sensors were placed in a Teflon sensing chamber with gas inlet and outlet. The VOC vapors were generated from a temperature-controlled bubbler by introducing dry air in organic solvents, which was then diluted to a desired concentration by dry air flow. The flow rates were controlled by mass flow controllers (5850E, Brooks Instrument, USA). The total flow rate was fixed at 500 ml/min during the measurements. The concentration of a given VOC was calculated by equation 1 as:[1]

$$\mathrm{C(ppm)} = 10^6 \times \left(\frac{P_s}{P} \times \frac{f}{f+F}\right) \quad (1)$$

where C is the concentration of the VOC in ppm; f and F are the flow rates (in sccm) of the bubbling air saturated with VOC and air as dilution gas, respectively; P is the total pressure (which is atmospheric pressure in our system); Ps is the saturated partial pressure (in mm of Hg) of the VOC obtained from the following Antoine equation (equation 2),[2] where T is the temperature, A, B, and C are Antoine coefficients. The Antoine coefficients and associated data for vapor pressure calculations of various VOCs are summarized in Supplementary Table 1.

$$\mathrm{Log}_{10} P_s = \left(A - \frac{B}{C+T}\right) \quad (2)$$

The relative humidity (RH) in the chamber was controlled by adjusting the flow ratios of dilution gas (air) and moisture, monitored with a commercial humidity sensor (HDC 2010, Texas Instruments).

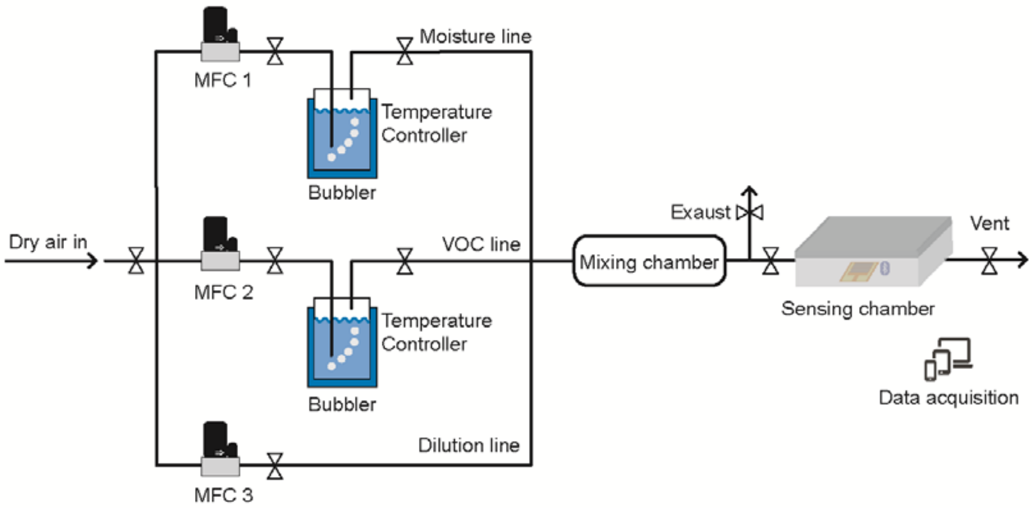

Supplementary Fig. 3 Schematic diagram showing the setup of a homemade gas-sensing system used to detect a variety of VOC gases.

Supplementary Table 1 Antoine coefficients of the various solvents used for vapor sensing experiments.[2]

Solvent	A	B	C	T_{min} (°C)	T_{max} (°C)
Ethanol	8.12875	1660.8713	238.131	–5.15	240.75
Methanol	8.08404	1580.4585	239.096	–15.99	199.45
Acetone	7.31742	1315.6735	240.479	–32.22	234.95
Hexane	6.98978	1216.9154	227.451	–24.29	92.12
Benzene	6.81432	1090.4312	197.146	–9.60	103.04
Toluene	7.13657	1457.2871	231.827	5.65	136.67

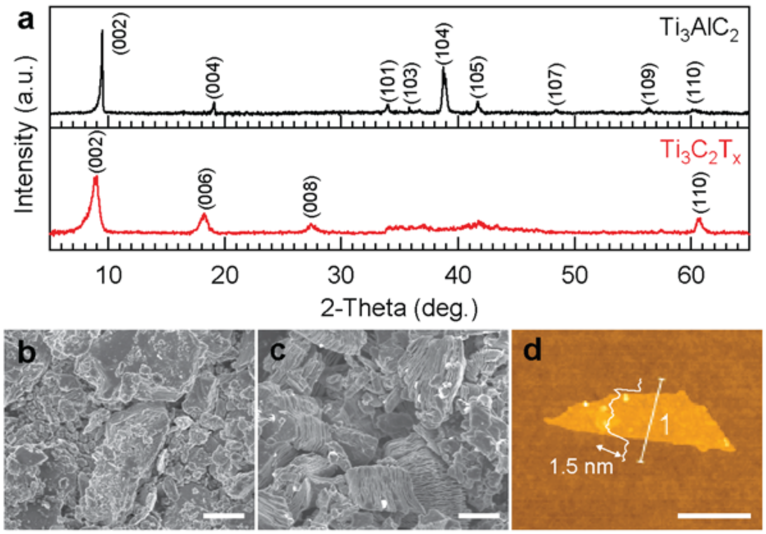

Supplementary Fig. 4 Verification of $Ti_3C_2T_x$ formation using XRD, SEM, and AFM imaging. **a** XRD patterns showing the transformation of Ti_3AlC_2 to $Ti_3C_2T_x$. SEM images of **b** Ti_3AlC_2 and **c** $Ti_3C_2T_x$ (scale bars, 2 μm). **d** AFM image of the delaminated $Ti_3C_2T_x$ nanosheet (scale bar, 200 nm). The intense peaks of Ti_3AlC_2 at ~39° are replaced by a broadened peak with low intensity, which corresponds to the elimination of Al interlayer atoms and formation of $Ti_3C_2T_x$.[3] The (002) diffraction peaks for Ti_3AlC_2 at 9.5° shifts to 9.0° for $Ti_3C_2T_x$, suggesting the introduction of surface functional group.[4] The XRD results are consistent with the SEM images with accordion-like structure observed in $Ti_3C_2T_x$.[5]

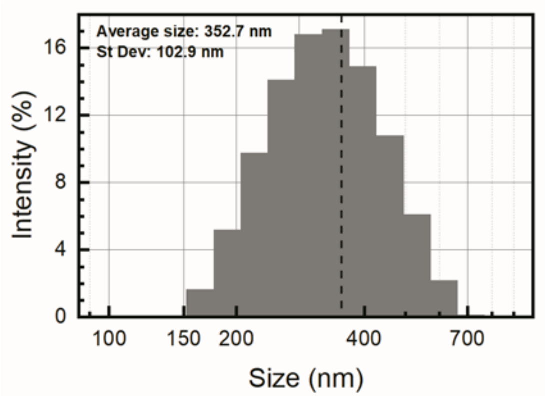

Supplementary Fig. 5 The size distribution of $Ti_3C_2T_x$/ WSe_2 nanohybrids measured by dynamic light scattering.

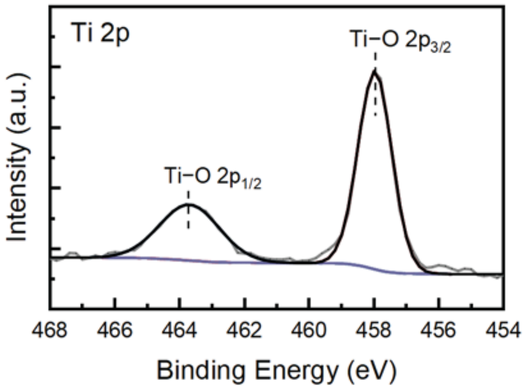

Supplementary Fig. 6 Ti 2p XPS spectrum of oxidized $Ti_3C_2T_x$ nanosheets showing only Ti−O peaks.

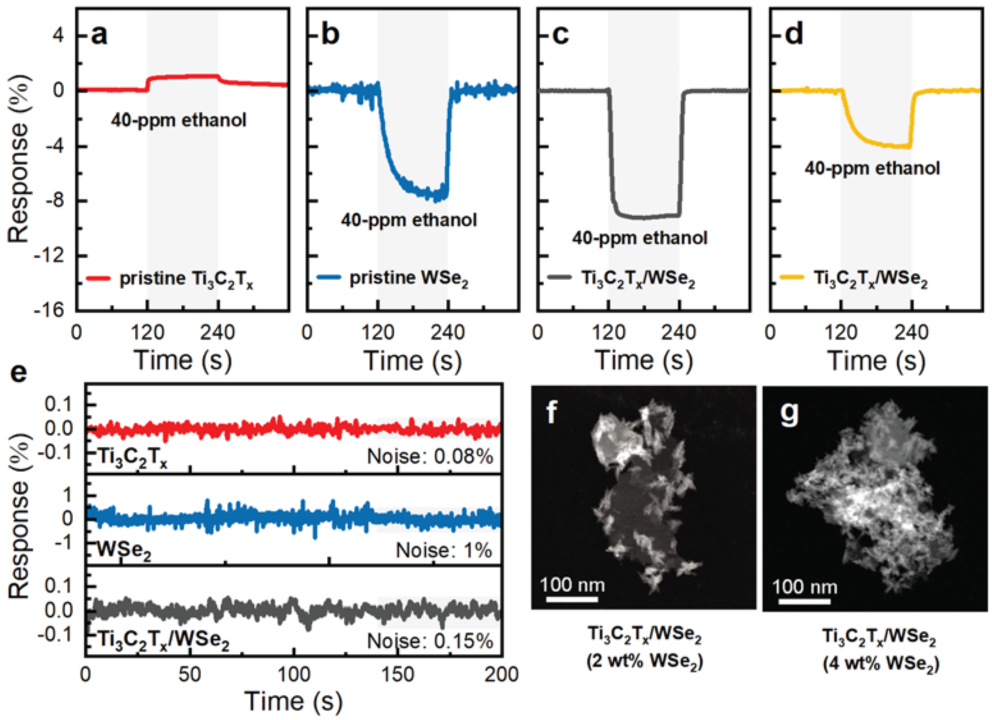

Supplementary Fig. 7 Sensing characteristics of WSe_2, $Ti_3C_2T_x$, and $Ti_3C_2T_x/WSe_2$ sensors. Comparison of room-temperature sensing responses of **a** pristine $Ti_3C_2T_x$, **b** WSe_2, **c** $Ti_3C_2T_x/WSe_2$ (2 wt%), and **d** $Ti_3C_2T_x/WSe_2$ (4 wt% WSe_2) upon exposure to 40-ppm ethanol. **e** electrical noise measurements. HAADF-STEM images of **f** $Ti_3C_2T_x/WSe_2$ (2 wt% WSe_2) and **g** $Ti_3C_2T_x$ (4% WSe_2). Scale bars: 100 nm.

Supplementary Note 2: Comparing Supplementary Fig. 7a–d shows that, upon exposure to ethanol, the $Ti_3C_2T_x$ sensor had a relatively small, positive response, while the WSe_2 and $Ti_3C_2T_x/WSe_2$ sensors yielded negative responses, indicating that the loading of WSe_2 nanoflakes (2 or 4 wt%) results in an alternation of sensing mechanisms of $Ti_3C_2T_x$ nanosheets, as discussed in the manuscript. Notably, the response of the $Ti_3C_2T_x/WSe_2$ (2 wt%) sensor is much higher and faster than that of the $Ti_3C_2T_x/WSe_2$ (4 wt%) sensor (compare Supplementary Fig 7c, d).

According to Supplementary Fig. 7e, electrical noise levels of WSe_2, $Ti_3C_2T_x$, and $Ti_3C_2T_x/WSe_2$ (2 wt%) were approximately 1%, 0.08%, and 0.15%, respectively. The highest noise level of the WSe_2 having high resistance nature is the hurdle that limits the development of high-performance room-temperature sensors. Notably, the individual $Ti_3C_2T_x$ with a higher electrical conductance has the lowest noise of 0.08%, but its response value is the lowest among the four sensors tested. In contract, the sensor based on $Ti_3C_2T_x/WSe_2$ (2 wt%) nanohybrids exhibits the highest response and very low electrical noise (0.15%), indicating that this hybrid material outperforms both individual WSe_2 and $Ti_3C_2T_x$ for VOC sensing. As revealed by the HAADF-STEM images in Supplementary Fig. 7e and f, an excess of WSe_2 concentration in the hybridization process causes the $Ti_3C_2T_x$ hosting matrix to cover with an excessive number of WSe_2 nanoflakes, resulting in blocking of the $Ti_3C_2T_x/WSe_2$ heterojunctions as the major gas reaction sites, thus further decreasing the sensor response.

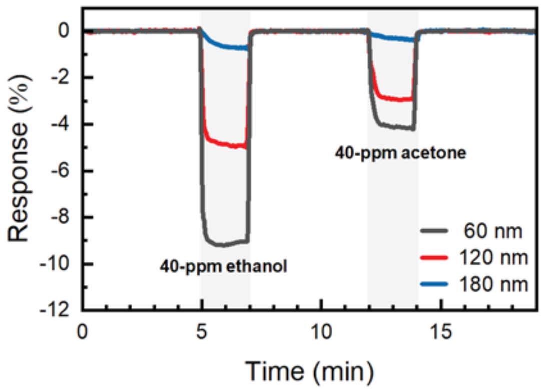

Supplementary Fig. 8 Responses of 60, 120, and 180-nm-thick $Ti_3C_2T_x/WSe_2$ electrode films towards 40 ppm of ethanol and acetone.

Supplementary Table 2 A summary of recent studies of sensing volatile organic compounds based on chemiresistive gas sensors.

Materials	Substrate	Method	Analyte	Concentration	Response (%)	Temperature	Response time	Ref
$Ti_3C_2T_x/WSe_2$	polyimide	solution	ethanol	1 −40 ppm	0.5 − 9.2 [a]	RT	9.7/6.6 s	this work
$Ti_3C_2T_x$	polyimide	solution	ethanol	100 ppm	11.5[a]	RT	–	6
WSe_2	SiO_2/Si	ALD	acetone	10 ppm	16.74[a]	RT	–	7
MoS_2	SiO_2/Si	solution	ethanol	3 ppm	1[a]	300 °C	–	8
MoS_2/WS_2	–	hydrothermal	ethanol	50 ppm	2[b]	RT	–	9
$Mo_{1-x}W_xS_2$	SiO_2/Si	solution	acetone	5 ppm	0.25[a]	RT	–	10
Ag/WS_2	SiO_2/Si	ALD	acetone	10 ppm	33[a]	100 °C	–	11
Au/MoS_2	PET	solution	acetone	10 ppm	15[a]	RT	2.7/1.8 min	12
Pt/WS_2	mica	hydrothermal	ethanol	500 ppm	9.3[a]	RT	–	13
TiO_2/MoS_2	Al_2O_3	hydrothermal	ethanol	50 ppm	10[b]	150 °C	–	14
SnO_2/MoS_2	Al_2O_3	hydrothermal	ethanol	50 ppm	50[b]	280 °C	–	15
rGO/WS_2	Al_2O_3	hydrothermal	ethanol	30 ppm	10[a]	33.5 °C	–	16
graphene/MoS_2	Al_2O_3	solution	methanol	10 ppm	1.6[a]	RT	3.5/3.7 min	17

[a]Response (%) = $|(I_g – I_0)|/I_0 \times 100$ or $|(R_g – R_0)|/R_0 \times 100$, [b]Response = I_g/I_0

Supplementary References

1. Behera, B., Chandra, S. A MEMS based acetone sensor incorporating ZnO nanowires synthesized by wet oxidation of Zn film. *J. Micromech. Microeng.* **25**, 015007 (2015).
2. Yaws, C. L., Satyro, M. A. The Yaws Handbook of Vapor Pressure. In: *The Yaws Handbook of Vapor Pressure (Second Edition)* (ed Yaws CL). Gulf Professional Publishing (2015).
3. Naguib, M., *et al.* Two-dimensional nanocrystals produced by exfoliation of Ti_3AlC_2. *Adv. Mater.* **23**, 4248–4253 (2011).
4. Dai, B., *et al.* Novel two-dimensional $Ti_3C_2T_x$ MXenes/nano-carbon sphere hybrids for high-performance microwave absorption. *J. Mater. Chem. C* **6**, 5690–5697 (2018).
5. Alhabeb, M., *et al.* Guidelines for synthesis and processing of two-dimensional titanium carbide ($Ti_3C_2T_x$ MXene). *Chem. Mater.* **29**, 7633–7644 (2017).
6. Lee, E., VahidMohammadi, A., Prorok, B. C., Yoon, Y. S., Beidaghi, M., Kim, D. J. Room temperature gas sensing of two-dimensional titanium carbide (MXene). *ACS Appl. Mater. Interfaces* **9**, 37184–37190 (2017).
7. Ko, K. Y., *et al.* Recovery Improvement for Large-Area Tungsten Diselenide Gas Sensors. *ACS Appl. Mater. Interfaces* **10**, 23910–23917 (2018).
8. Kim, Y. H., *et al.* Ultrasensitive reversible oxygen sensing by using liquid-exfoliated MoS2 nanoparticles. *J. Mater. Chem. A* **4**, 6070–6076 (2016).
9. Ikram, M., *et al.* Fabrication and characterization of a high-surface area MoS_2@WS_2 heterojunction for the ultra-sensitive NO_2 detection at room temperature. *J. Mater. Chem. A* **7**, 14602–14612 (2019).
10. Yang, K., *et al.* Composition- and phase-controlled synthesis and applications of alloyed phase heterostructures of transition metal disulphides. *Nanoscale* **9**, 5102–5109 (2017).
11. Ko, K. Y., *et al.* Improvement of gas-sensing performance of large-area tungsten disulfide nanosheets by surface functionalization. *ACS Nano* **10**, 9287–9296 (2016).
12. Chen, W. Y., Yen, C. C., Xue, S., Wang, H., Stanciu, L. A. Surface functionalization of layered molybdenum disulfide for the selective detection of volatile organic compounds at room temperature. *ACS Appl. Mater. Interfaces* **11**, 34135−34143 (2019).
13. Ouyang, C., *et al.* Two-dimensional WS_2-based nanosheets modified by Pt quantum dots for enhanced room-temperature NH_3 sensing properties. *Appl. Surf. Sci.* **455**, 45–52 (2018).
14. Zhao, P. X., *et al.* One-dimensional MoS_2-decorated TiO_2 nanotube gas sensors for efficient alcohol sensing. *J. Alloy. Compd.* **674**, 252–258 (2016).
15. Yan, H. H., Song, P., Zhang, S., Yang, Z. X., Wang, Q. Dispersed SnO_2 nanoparticles on MoS_2 nanosheets for superior gas-sensing performances to ethanol. *RSC Adv.* **5**, 79593–79599 (2015).
16. Wang, X., *et al.* Reduced graphene oxide hybridized with WS_2 nanoflakes based heterojunctions for selective ammonia sensors at room temperature. *Sens. Actuator B-Chem.* **282**, 290–299 (2019).
17. Zhang, S.-L., Yue, H., Liang, X., Yang, W.-C. Liquid-phase co-exfoliated graphene/MoS_2 nanocomposite for methanol gas sensing. *J. Nanosci. Nanotechnol.* **15**, 8004–8009 (2015).
